# Myzorhynchus series of *Anopheles* mosquitoes as potential vectors of *Plasmodium bubalis* in Thailand

**DOI:** 10.1038/s41598-022-09686-9

**Published:** 2022-04-06

**Authors:** Yudhi Ratna Nugraheni, Apinya Arnuphapprasert, Trang Thuy Nguyen, Duriyang Narapakdeesakul, Hoang Lan Anh Nguyen, Juthathip Poofery, Osamu Kaneko, Masahito Asada, Morakot Kaewthamasorn

**Affiliations:** 1 The International Graduate Program of Veterinary Science and Technology (VST), Faculty of Veterinary Science, Chulalongkorn University, Bangkok, 10330 Thailand; 2 Veterinary Pathobiology Graduate Program, Faculty of Veterinary Science, Chulalongkorn University, Bangkok, 10330 Thailand; 3 Veterinary Parasitology Research Unit, Faculty of Veterinary Science, Chulalongkorn University, Bangkok, 10330 Thailand; 4 Department of Protozoology, Institute of Tropical Medicine (NEKKEN), Nagasaki University, Nagasaki, 852-8523 Japan; 5 National Research Center for Protozoan Diseases, Department of Global Cooperation, Research Unit for Global Infection Control, Obihiro University of Agriculture and Veterinary, Obihiro, 080-8555 Japan; 6 Department of Parasitology, Faculty of Veterinary Medicine, Universitas Gadjah Mada, Yogyakarta, 55281 Indonesia

**Keywords:** Parasitology, Entomology, Ecology, Evolution, Microbiology, Molecular biology, Zoology

## Abstract

Ungulate malaria parasites and their vectors are among the least studied when compared to other medically important species. As a result, a thorough understanding of ungulate malaria parasites, hosts, and mosquito vectors has been lacking, necessitating additional research efforts. This study aimed to identify the vector(s) of *Plasmodium bubalis*. A total of 187 female mosquitoes (133 *Anopheles* spp., 24 *Culex* spp., 24 *Aedes* spp., and 6 *Mansonia* spp. collected from a buffalo farm in Thailand where concurrently collected water buffalo samples were examined and we found only *Anopheles* spp. samples were *P. bubalis* positive. Molecular identification of anopheline mosquito species was conducted by sequencing of the PCR products targeting *cytochrome c oxidase subunit 1* (*cox1*), *cytochrome c oxidase subunit 2* (*cox2*), and internal transcribed spacer 2 (*ITS2*) markers. We observed 5 distinct groups of anopheline mosquitoes: Barbirostris, Hyrcanus, Ludlowae, Funestus, and Jamesii groups. The Barbirostris group (*Anopheles wejchoochotei* or *Anopheles campestris*) and the Hyrcanus group (*Anopheles peditaeniatus*) were positive for *P. bubalis*. Thus, for the first time, our study implicated these anopheline mosquito species as probable vectors of *P. bubalis* in Thailand.

## Introduction

Malaria parasites of the genus *Plasmodium*, particularly in most of the medically important species, have undergone intensive studies, and they are well manageable as a result. Historically, descriptions of *Plasmodium* species infecting even-toed ungulates (order Artiodactyla), on the other hand, have appeared intermittently in literature (see review in Templeton et al.^1^). Among these, *Plasmodium bubalis* was discovered in Murrah buffalo (Bovidae: *Bubalus bubalis*) in India^2^ and was later reported in water buffaloes in several other countries (see for example Templeton et al.^3^; Kandel et al.^4^). *Plasmodium traguli* was found in mousedeer (Tragulidae: *Tragulus javanicus*) in Malaysia decades ago and has not appeared in literature since^5,6^. *Plasmodium caprae* was first recorded in African goats (Bovidae: *Capra aegagrus hircus*)^7^ and more recently in several countries outside Africa, including Thailand^3,8^. Among the various ungulate malaria parasites described thus far, at least three are endemic in Southeast Asia, suggesting the presence of mosquito vectors in this region. Most of the first discoveries of ungulate malaria occurred prior to the implementation of PCR in 1980, and thus vector identification efforts relied solely on morphological investigations. As a result, a comprehensive picture of these taxa’s transmission cycle could not be drawn.

According to Rattanarithikul et al.^9^ and the Walter Reed Biosystematics Unit^10^, at least 464 mosquito species have been recorded in Thailand, with 83 of these belonging to the Anophelinae subfamily. It is not surprising that vector studies on malaria of medical importance have gained greater attention and achieved greater accomplishments than others^11,13^. The majority of anopheline species in Southeast Asian countries are cryptic species complexes^14–16^. The Barbirostris complex, for example, includes at least six species, five of which are found in Thailand^17,18^. Misidentification is a common pitfall in vector studies, particularly when dealing with species complexes^15^. Such deceptive results could have an impact on vector control programs or mislead subsequent studies^19–21^.

Despite several limitations and difficulties, a study of mosquitoes feeding on infected mousedeer in Malaysia resulted in the successful incrimination of *Anopheles umbrosus* and *Anopheles letifer* as probable vectors of *P. traguli*^22^. After decades of inactivity, sporozoites of unknown malaria parasites were isolated from the salivary glands of *Anopheles gabonensis* and *Anopheles obscurus* in Gabon^23^. The cytochrome b sequences isolated from these sporozoites share the same clade with *Plasmodium* DNA detected from African ungulates. *Plasmodium* sporozoites were observed in the salivary glands of *Anopheles punctipennis* in a separate study conducted in the United States of America. Phylogenetic analysis revealed that their sequences were related to *Plasmodium* from white-tailed deer (Cervidae: *Odocoileus virginianus*)^24^. However, little is known about the vectors of *P. bubalis* and *P. caprae*, both of which are endemic in Southeast Asia. *Anopheles minimus* has been suspected of transmitting *P. bubalis*^25^. However, incrimination of this mosquito species remains controversial without clear evidence to support it. In Thailand and other countries, very limited research works on *P. bubalis* and its vector have been published^3,4^. Recently, a high prevalence of *P. bubalis* infection has been reported in Thailand; however, no information on the transmission or the probable vector has been provided^26^. We hypothesized that the mosquito vectors of *P. bubalis* are likely endemic species in the country. Therefore, we conducted this study aiming to identify the anopheline mosquito species transmitting *P. bubalis* in Thailand.

## Results

### *P. bubalis* detected in buffalo blood samples on a farm

Previous investigation in Thailand revealed that 35% of buffaloes on a farm located in Chachoengsao Province were infected with *P. bubalis*^26^. Thus, this study selected the same farm to identify the vector mosquitoes of *P. bubalis*. A total of 90 buffalo blood samples were collected in June 2020 (n = 45) and November 2021 (n = 45) from the farm. Mosquitoes were captured then underwent PCR screening for *P. bubalis* infection using primers targeting the *cytb* gene. Two buffalo blood samples (IDs THBuff20_37 and THBuff20_39) from the 2020 collection were positive (4.4%), indicating that *P. bubalis* infection occurred on this farm when mosquito samples were collected. In the 2021 collection, no blood or anopheline mosquito samples were positive.

### Species composition of mosquitoes collected from buffalo farm by morphology

**Figure 1:**
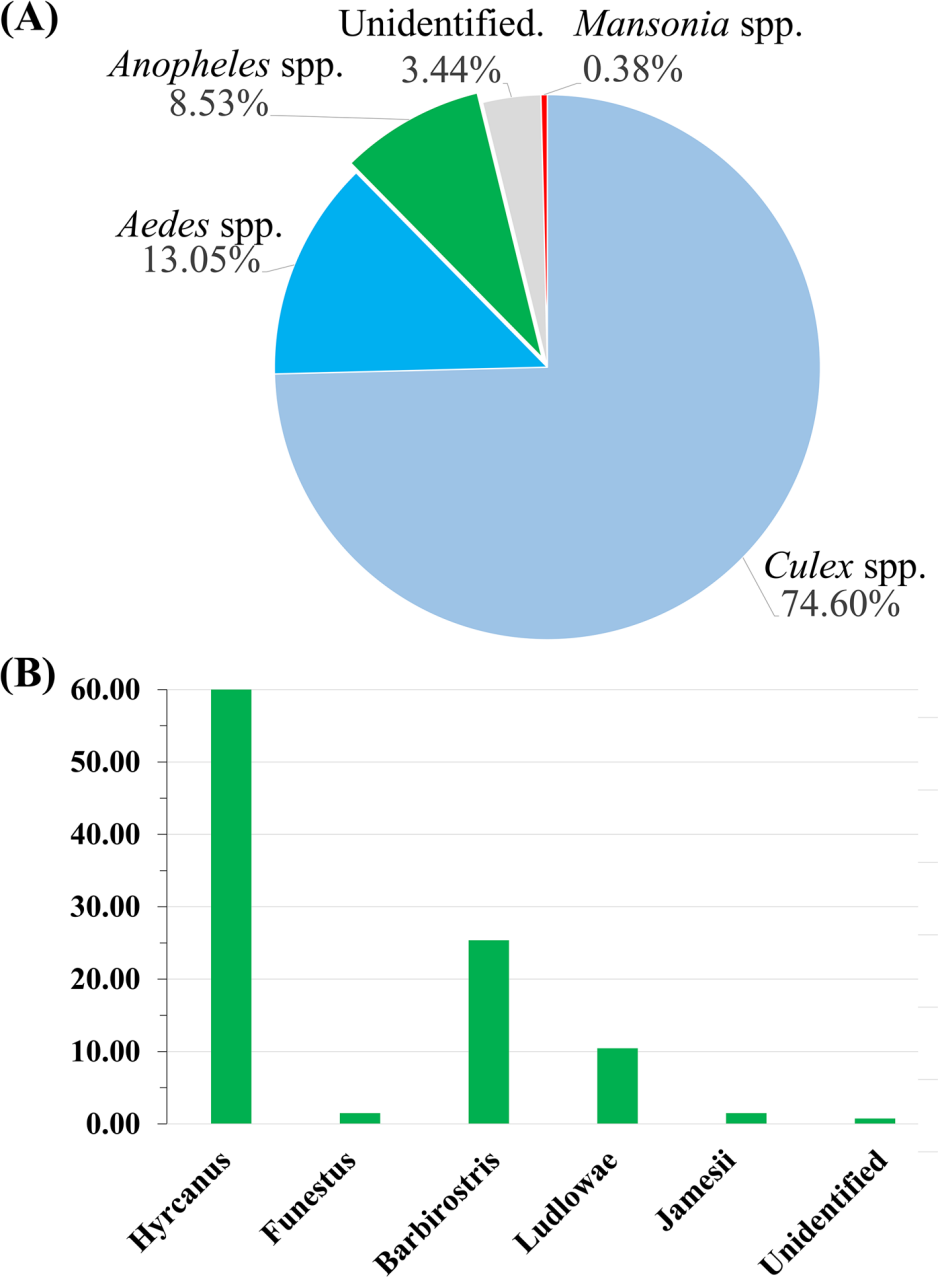
Chart illustrating the percentage of mosquitoes, according to morphological identification. (**A**) Percentages of each genus of mosquitoes collected in this study. (**B**) *Anopheles* mosquito groups.

A total of 1,571 female mosquitoes were collected from a farm in Chachoengsao. Morphological examination indicated that anopheline mosquitoes accounted for 8.53% (n = 134), while *Culex* spp. accounted for 74.6% (n = 1172), *Aedes* spp. accounted for 13.05% (n = 205), *Mansonia* spp. accounted for 0.38% (n = 6), and unidentifiable due to body part destruction accounted for 3.44% (n = 54) (Fig. 1A). Among 134 anopheline mosquitoes, 5 different *Anopheles* groups were identified including Barbirostris, Hyrcanus, Funestus, Ludlowae, and Jamesii groups; 1 mosquito was unable to be identified in any group due to missing wings and legs (Fig. 1B).

### Identification of *P. bubalis* DNA from mosquito salivary gland samples

For a total of 133 identified anopheline mosquitoes, salivary glands with the head and thorax were carefully separated from the rest of the mosquitoes’ bodies, then the salivary glands and midguts were stained with 0.1% mercurochrome dye and examined under a microscope. However, no oocysts and sporozoites were found. Then, one to three samples consisting of the salivary glands, head, and thorax were combined based on the group and, finally, 51 pooled samples were prepared (Table 1). DNA was extracted from the samples and PCR screening was performed for *Plasmodium cytb*, *18S rRNA*, and *cox1* genes. The number of each pool was as follows: Barbirostris group (n = 35, 23 pools), Hyrcanus group (n = 81, 19 pools), Ludlowae group (n = 14, 7 pools), Funestus group (n = 1, 1 pool), and Jamesii group (n = 2, 1 pool). Out of 51 pools of anopheline mosquitoes, 3 pools were PCR positive for *Plasmodium*. These samples were the Barbirostris group (IDs THMosqBuff20_P6_3, THMosqBuff20_P8_2) and Hyrcanus group (ID THMosqBuff20_P20_3) (Table 1). The minimum infection rates (MIR) were 5.7% (0.015–0.186) in the Barbirostris group mosquito and 2.5% (0.004–0.128) in the Hyrcanus group mosquito (Table 2). Additionally, for those of non-anopheline mosquitoes, a total of 22 pools (*Culex* spp. n = 24, 8 pools); *Aedes* spp n = 24, 8 pools; and *Mansonia* spp. n = 6, 6 pools) were tested. *Plasmodium bubalis* was not detected in any *Culex* spp., *Aedes* spp., or *Mansonia* spp. pools.

**Table 1:** Summary of *P. bubalis*’s PCR screening results of anopheline mosquitoes collected from the buffalo farm.

Sampling sites	Group	No. collected	No. of pools	No. of positive pools			No. of pools sequenced for mosquito genes and determined species name
				*cytb*	*18S rRNA*	*cox1*	
Chachoengsao	Barbirostris	35	23	2	2	2	17 (*An. campestris* or *An. wejchoochotei*)
	Hyrcanus	81	19	1	1	1	15 (*An. peditaeniatus*), 1 (*An. sinensis*)
	Funestus	1	1	0	0	0	1 (*An. varuna*)
	Ludlowae	14	7	0	0	0	6 (*An. vagus*)
	Jamesii	2	1	0	0	0	1 (*An. pseudojamesi*)
Total		133	51	3	3	3	41

**Table 2:** Minimum infection rates of *Plasmodium* in collected mosquitoes.

Species	Total no. mosquitoes	Pool size (range)	No. tested	No. positive pools	MIR (%) (95% CI)
*An. campestris* or *wejchoochotei*	35	1–3	35	2	5.7 (0.015–0.186)
*An. peditaeniatus*	81	1–3	52	1	2.5 (0.004–0.128)

Analysis by the BLASTN program using *cytb* and *cox1* sequences obtained from 3 pools against non-redundant nucleotide collection revealed that they were 100% identical to *P. bubalis* type I (accession no. LC090213). Analysis by the BLASTN program using putative *P. bubalis*’s *18S rRNA* sequences did not identify any sequences in the database with 100% identity. The maximum identity was 92% with *18S rRNA* sequences of *Plasmodium falciparum* (accession no. LR131366) as well as those of other *Plasmodium* species. Because no *18S rRNA* sequences derived from any ungulate malaria parasites were available in the GenBank™ database, we used two buffalo-derived samples (IDs THBuff20_37 and THBuff20_39) for PCR-amplification with the same universal primers for *Plasmodium* 18S rRNA and sequences were determined. Sequences derived from 3 mosquito samples showed 100% identity with the sequences from 2 buffalo samples, further supporting the presence of *P. bubalis* in the mosquitoes.

**Figure 2:**
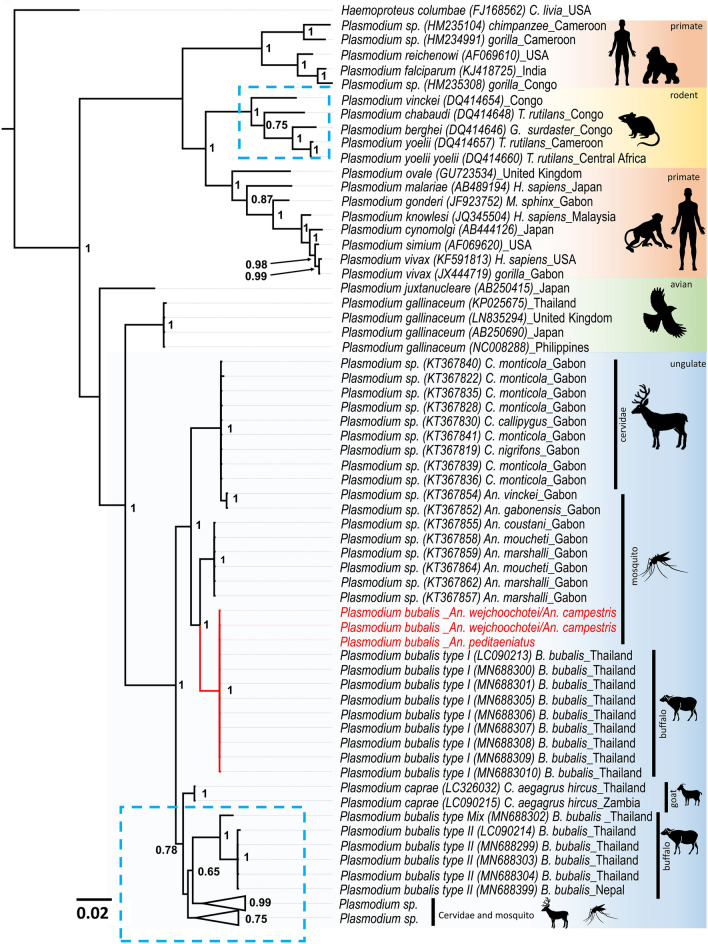
Phylogenetic positions of *Plasmodium* detected from *Anopheles* mosquitoes in this study. The phylogenetic tree was inferred by Bayesian inference method using partial *cytb* sequences (789 bp). *Haemoproteus columbae* was used to root all sequences. At the nodes, Bayesian posterior probabilities (PP ≥ 0.65) are indicated. *Plasmodium* sequences obtained in this study are highlighted in red. The length for the substitutions/site (0.02) is indicated.

Phylogenetic analyses using the *cytb* (789 bp), *cox1* (254 bp), and *18S rRNA* (351 bp) genes revealed that *Plasmodium* sequences from this study belong to the same cluster as *P. bubalis* type I isolates previously reported from Thailand (Fig. 2, Suppl. Figure 2, Suppl. Figure 3). The current findings indicated that all *Plasmodium* sequences obtained from mosquitoes in this study were *P. bubalis* type I.

### Molecular identification of anopheline mosquitoes collected from a buffalo farm

To identify the species of anopheline mosquitoes collected from buffalo farms by molecular analysis, *cox1*, *cox2*, and *ITS2* gene sequences were determined for three *Plasmodium*-positive *Anopheles* mosquito pools, as well as 15 additional *Plasmodium*-negative pools in this study. The obtained sequences were initially assessed by the BLASTN program against a non-redundant nucleotide collection for species identification. Based on the sequence of DNA barcoding region for mosquito identification, several studies have suggested an evolutionary divergence of 2–3% as a threshold for intraspecific variation^27–29^. Thus, sequences with the highest identity (minimum ≥ 97%) are listed in Supplementary Table 2. BLASTN analysis of some *cox1* sequences obtained in this study was unable to reach this threshold, indicating the limitation of this approach due to the insufficient collection of mosquito sequences in the database. Nonetheless, analysis of all 3 genes of 1 Funestus group pool was matched to *An. varuna*. All 3 gene sequences of one Ludlowae group mosquito hit *An. vagus*. *An. peditaeniatus* was hit by two Hyrcanus group pools with all 3 gene sequences including a *P. bubalis* sequence-positive pool (THMosqBuff20_P20_3), and by one Hyrcanus group pool with *cox2* and *ITS2* sequences. *An. pseudojamesi* was hit by one Jamesii group pool.

The *ITS2* sequences of 9 pools of Barbirostris group mosquitoes showed 98.8–100% identity to *An. campestris* or *An. wejchoochotei*. The *Cox2* sequences of 9 pools of Barbirostris group mosquitoes showed 99–100% identity to *An. campestris*. However, there were no *An. wejchoochotei cox2* sequences available in the database, which limited the assessment of the *cox2* sequence with *An. wejchoochotei*. BLASTN search using 8 *cox1* sequences (1,416 bp) hit *An. donaldi* with ~ 97% identity and one *cox1* sequence (333 bp) showed 99.1% identity to *An. campestris*. Because all *An. wejchoochotei cox1* sequences deposited in the database are much shorter than the 8 sequences in this study, we aligned our *cox1* sequences with *An. wejchoochotei cox1* sequences (AB971335, AB971336, AB971337, AB971338, AB971339, and AB971340) from the morphologically well-described samples^18^ and found they were matched with > 99% identity (Supplementary Fig. 1). Because *An. wejchoochotei* sequences were reported in 2015 from morphologically defined samples^18^, and the identities of "*An. campestris*" from which DNA sequences were deposited to the database before this report were not clear, it was impossible to distinguish *An. campestris* and *An. wejchoochotei* molecularly at the time. Thus, we concluded that *P. bubalis*-positive anopheline mosquitoes from the Barbirostris group (THMosqBuff20_P6_3 and THMosqBuff20_P8_2) were either *An. campestris* or *An. wejchoochotei*; one from the Hyrcanus group (THMosqBuff20_P20_3) was *An. peditaeniatus.*

## Discussion

The current study aimed to identify potential *P. bubalis* vectors in Thailand. *An. wejchoochotei* or *An. campestris, An. peditaeniatus, An. varuna, An. vagus*, and *An. pseudojamesi* were molecularly confirmed on a farm where *P. bubalis* was detected from water buffaloes, and *P. bubalis* DNA sequences were detected from *An. wejchoochotei* or *An. campestris,* and *An. peditaeniatus.* According to Rattanarithikul et al.^9^ and the Walter Reed Biosystematics Unit^10^, all of these anopheline mosquitoes have previously been recorded in districts throughout Thailand as well as across Southeast Asian countries. *An. wejchoochotei* is found in Thailand and Cambodia, whereas *An. peditaeniatus* can be found in Thailand, Cambodia, Indonesia, Malaysia, Myanmar, the Philippines, and Vietnam^9,17,18,30–33^. *An. wejchoochotei* and *An. peditaeniatus* were recently found to harbor human *Plasmodium* species in Cambodia^13^.

In this study, we detected *P. bubalis*’s DNA in salivary gland samples, but oocysts and sporozoites were not observed under a microscope. This was most likely due to the low infection rate of the parasite in the water buffaloes, which resulted in a low parasite burden in the mosquitoes^3^. *P. traguli* oocysts and sporozoites have been discovered in *An. umbrosus* and *An. letifer* by microscopic examination in a historic mousedeer study in Malaysia^22^. The successful observation of *P. traguli* in mosquitoes may be due to a relatively higher infection rate in mousedeers than *P. bubalis* in water buffaloes because the *P. traguli* detection rate in the mousedeer blood samples was high (≥ 37%)^22^.

Furthermore, nucleotide sequence analysis using Bayesian Inference (BI) confirmed that *Plasmodium* parasites isolated from *An. wejchoochotei* or *An. campestris* and *An. peditaeniatus* in this study were genetically identical and were grouped to previously described *P. bubalis* type I isolated from buffaloes^26^, suggesting that these mosquito species were plausible vectors for *P. bubalis*.

Taai and Harbach^18^ described *An. wejchoochotei* for the first time, while Reid^34^ recorded *An. campestris* in 1962. It should be noted that mosquitoes from Thailand that have since been identified as *An. wejchoochotei* were initially referred to as *An. campestris*-like by Harrison and Scanlon^35^ due to their resemblance to *An. campestris*. Both are members of the Barbirostris complex group and cannot be distinguished solely by the morphology of the adult mosquitoes; morphological information of the larva is required. Previous research suggested that *cox1, cox2,* and *ITS2* are reliable genetic markers for distinguishing cryptic species within the complex group of anopheline mosquitoes^36,37^. A recent study in Sulawesi, Indonesia, used approximately 700 bp of the *cox1* gene to distinguish members of mosquito species complexes^38^. Furthermore, *cox1* and *ITS2* sequences have been used to identify cryptic mosquito species^39^. Based on the *cox1* barcode region, an evolutionary divergence of 0.5% (range 0.0–3.9%) was proposed as a threshold for intraspecific variation^28^. Consequently, we carried out an investigation into the *cox1*, *cox2*, and *ITS2* markers of anopheline mosquitoes in this study. We found that sequences from *Plasmodium*-positive mosquitoes (THMosqBuff20_P6_3 and THMosqBuff20_P8_2) showed high similarity with either *An. campestris* or *An. wejchoochotei* sequences in the GenBank™ database. The conflicting species discrimination of the previously deposited sequences between *An. campestris* and *An. wejchoochotei* (formerly, *An. campestris*-like) will be solved by molecular analysis of the morphologically confirmed *An. campestris* samples in the future.

The Barbirostris and Hyrcanus groups belong to the Myzorhynchus series of *Anopheles* mosquitoes, which contains most vectors of human malaria except for *An. punctipennis*, which belongs to the Anopheles series. *An. umbrosus* and *An. letifer*, suspected vectors of *P. traguli*, and *An. gabonensis* and *An. obscurus*, the vectors of African ungulate malaria parasites, also belong to the Myzorhynchus series. Thus, Myzorhynchus series mosquitoes appear to have a dominant role in the transmission of ungulate malaria parasites.

## Conclusions

*An. wejchoochotei* or *An. campestris* and *An. peditaeniatus* were identified as vectors of *P. bubalis* type I.

## Methods

### Study site, mosquito collection, dissection, and DNA extraction

**Figure 3:**
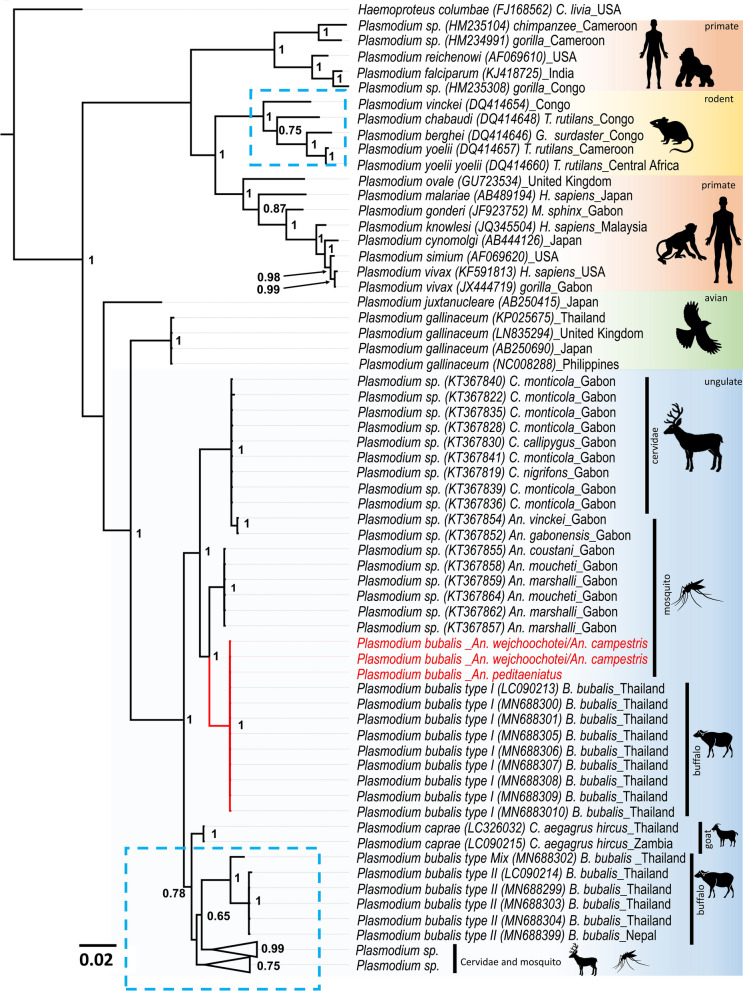
(**A**) Map depicting a buffalo farm in Chachoengsao for sample collection in Thailand. (**B**) The landscape of mosquito sampling sites in a buffalo farm in Chachoengsao. The images were obtained and modified from Google Earth Pro version 7.3.4.8248. The red triangle indicates blood sample collection sites, while the yellow triangle indicates mosquito sampling sites.

This study was conducted on a buffalo farm in Chachoengsao province of Thailand (Fig. 3A). To investigate mosquito composition and identify the probable vector of *P. bubalis*, we carried out a survey of Murrah dairy buffaloes in Chachoengsao Province (13°28′53.98"N 101°27′35.23"E) for 14 consecutive nights in June 2020 and 2 nights in November 2021. The Murrah dairy buffalo farm is located 1 km away from the Nong Mai Kaen community. The area is surrounded by rubber trees with small ponds to wallow the water buffaloes (Fig. 3B).

CDC light traps with dry ice were set overnight at less than 1.5 m above ground level. Peripheral nets were placed surrounding the buffalo stable. Mosquitoes on the peripheral net were captured from 7.30 PM to 11.30 PM using tube aspirators (10 mm in diameter × 200 mm in length). The mosquitoes were then brought to the laboratory for morphological and molecular analysis. All anopheline mosquitoes were identified into group/species levels using taxonomic keys^9,40^, while non-anopheline mosquitoes were identified up to only genus level according to the pictorial identification key of important disease vectors in the WHO Southeast Asia^41^. Anopheline mosquitoes were carefully dissected within three days after collection to obtain the salivary glands of each mosquito. A 26G and ½ inch-long sterile needle was used to dissect individual mosquitoes, which was changed after each dissection to prevent cross-contamination. In addition, 0.1% mercurochrome dye was used to stain oocysts on the midgut wall and sporozoites in the salivary glands, and samples were examined under a microscope at 1,000-times magnification. Salivary glands, which were still attached to the head and thorax, were kept in 0.2 mL of 1 × PBS at 4 °C for further DNA extraction for mosquito species identification and malaria parasite detection.

DNA samples from mosquitoes were extracted using NucleoSpin® Tissue (Macherey–Nagel, Düren, Germany) according to the manufacturer’s guidelines with a minor modification in the elution step (elution volume reduced to 30 μL). Previous studies suggested that it is possible to detect higher infectivity in mosquito pool samples^42,43^. Thus, adult female mosquitoes were grouped based on their morphology. Mosquito pools were made following morphological identification and were subsequently confirmed by molecular identification. Each pool was made up of one to three mosquitoes from the same groups depending on sample availability.

### Blood collection from buffaloes, DNA extraction, and microscopic examination

To evaluate the malaria infection status in buffaloes, we carried out a survey of Murrah dairy buffaloes on a farm in Chachoengsao in June 2020 and November 2021, during which mosquitoes were captured (n = 45 and n = 45, respectively). These blood samples were drawn from the jugular vein using 21G needles and BD vacutainers containing acid citrate dextrose (ACD). It should be noted that *P. bubalis* have been detected from buffaloes on this farm in our previous surveys^3,26^. DNA was extracted as described above.

### Anopheline mosquito’s *cox1*, *cox2*, and *ITS2* gene amplification

Three genes of anopheline mosquito comprising *cox1, cox2,* and *ITS2* were amplified by PCRs using KOD FX Neo Polymerase (Toyobo, Japan) according to the manufacturer's protocol. The AnplCOXIF(5’-GGATCCCTTCAGCCATTTAATCGCG-3’) and AnplCOXIR primers (5’-TCGAGCTTAAATTCATTGCACTAATCTGCC-3’) were designed to amplify the *cox1* region with 1,584 bp-long products. The *Cox2* region was amplified by Anplcox2F-Anplcox2R primers (5’-GGATCCAGATTAGTGCAATGAATTTAAGC-3’) and (5’-CTGCAGGATTTAAGAGATCATTACTTGC-3’) to generate a total of 792 bp-long products. For the *ITS2* region, PCR amplification was carried out using ITS2A and ITS2B primers, as previously described^44^. The PCR product size of the *ITS2* region varied depending on the mosquito group (~ 1,500 bp for Barbirostris complex, ~ 562 bp for Hyrcanus, ~ 697 bp for Ludlowae, ~ 518 bp for Funestus, and ~ 555 bp for Jamesii).

### PCR detection of *Plasmodium’*s *cytb*, *18S rRNA*, and *cox1* genes

DNA samples from buffalo blood underwent nested PCR screening for *Plasmodium* using primers targeting *cytb* gene DW2 (5’-TAATGCCTAGACGTATTCCTGATTATCCAG-3’) and DW4 (5’-TGTTTGCTTGGGAGCTGTAATCATAATGTG-3’) as the outer primers and NCYBINF (5’-TAAGAGAATTATGGAGTGGATGGTG-3’) NCYBINR (5’-CTTGTGGTAATTGACATCCA-ATCC-3’) for the inner primers, as previously described^45^. Subsequently, *Plasmodium*-positive samples were further confirmed using primer sets targeting the *18S rRNA* and *cox1* genes. The first amplification of the *18S rRNA* gene was carried out using *Plasmodium* universal primers, rPLU5 (5’-CCTGTTGTTGCCTTAAACTTC-3’) and rPLU6 (5’-TTAAAATTGTTGCAGTTAAAACG-3’), as previously described by Snounou et al.^46^. New inner primers were designed based on the conserved region of the *18S rRNA* gene among the genus PlaSSUF1 (5’-CTTAGTTACGATTAATAGGAGTAG-3’) and PlaSSUR1 (5’-TCCTACT-CTTGTCTTAAACTAG-3’) for forward and reverse directions, respectively, for the second amplification. In addition, PCR targeting the *Plasmodium*’s *cox1* gene was conducted using the following primers: Cox1-F3-2 (5’-ATTATGTAATTGCACATTTCCATTTTG-3’) and Pbucox1-4B3 (5’-CCAAATAAAGTCATTGTWGAACC-3’). Each PCR amplification was carried out in a reaction volume of 12.5 μL, consisting of 2 × PCR buffer KOD FX Neo, 2.0 mM of dNTP, 0.4 μM of each primer, 1.0 Unit of KOD FX Neo DNA Polymerase (Toyobo, Japan), 1 μL genomic DNA as a template, and additional sterile distilled water up to 12.5 μL. The cycling conditions and product size of each PCR assay are described in Supplementary Table 1. Subsequently, 5 μL of PCR products were run on 1.5% agarose gel electrophoresis before being stained by Red Safe (Intron Biotechnology, Korea) and visualized under a UV transilluminator. The PCR products of positive samples were scaled up to 50 μL for purification and sequencing. Gel purification was carried out using NucleoSpin® Gel and PCR clean up (Macherey–Nagel, Düren, Germany) according to the manufacturers' protocols. Purified PCR products were sequenced in both directions. DNA samples extracted from mosquitoes were subjected to PCR screening for *P. bubalis* in the same way as mentioned in blood samples. Additionally, *Plasmodium*’s *cytb*-positive samples underwent PCR confirmation using primers targeting the *18S rRNA* and *cox1* genes, which were subsequently subjected to sequencing.

### Sequence analyses

The chromatogram files of all target genes were edited manually using BioEdit software version 7^47^. Low-quality sequences were excluded, resulting in a total of 41 mosquito pools being used for molecular analysis of each gene. Once the alignment was completed, sequences were compared to published sequence data in the GenBank™ database using the BLASTN program. The alignment of multiple sequences obtained from this study and additional sequences from the GenBank™ were made using the ClustalW via BioEdit version 7.

The ClustalW implemented in BioEdit version 7 was used to align sequences obtained in this study and additional sequences from GenBank™ database. MrBayes v3.2.750 was used to create phylogenetic trees using the Bayesian Inference (BI) method and the Markov chain Monte Carlo method. BI phylogenetic analysis was performed using two independent runs of four chains, each for 10 million generations. As a result of burn-in, the first 25% of trees were discarded. Tracer v1.751 was used to assess the mixing and convergence of runs, as well as effective sample sizes (EES > 200). FigTree v1.4.4 was used to visualize the trees (available at http://tree.bio.ed.ac.uk/software/figtree/).

### Statistical analysis

To evaluate the infection rate of positive mosquitoes, the minimum infection rate (MIR) was calculated for each species in which *Plasmodium* DNA was detected. If *Plasmodium* was detected from a mosquito pool, it was assumed that the pools contained at least one infected mosquito. Therefore, MIR was calculated as (number of positive pools/total number of analyzed mosquitoes) × 100, as previously described^48,49^. The MIR was calculated using the Wilson confidence interval method for binomial proportions, and the results were expressed as a percentage with a 95% confidence interval (CI).

### Ethics statement and biosafety

This study has been reviewed and approved by Chulalongkorn University Animal Care and Use Committee (Approval No. 1931027). All protocol in this study was performed according to the Institutional Biosafety Committee of Chulalongkorn University (No. 2031033).

## Supplementary Information

Supplementary Legends.

Supplementary Figure 1.

Supplementary Figure 2.

Supplementary Figure 3.

Supplementary Table 1.

Supplementary Table 2.

## Data Availability

All data in this article are available. Nucleotide sequences obtained in the present study were deposited in the GenBank™ database under the following accession numbers: OK338063, OL627356-57, OL672204-05 (*P. bubalis*’s *cox1*), OL624705-09 (*P. bubalis*’s *18S rRNA*), and OL672206-09 (*P. bubalis*’s *cytb*).
