# Publisher Correction: Myzorhynchus series of *Anopheles* mosquitoes as potential vectors of *Plasmodium bubalis* in Thailand

**DOI:** 10.1038/s41598-022-10860-2

**Published:** 2022-04-20

**Authors:** Yudhi Ratna Nugraheni, Apinya Arnuphapprasert, Trang Thuy Nguyen, Duriyang Narapakdeesakul, Hoang Lan Anh Nguyen, Juthathip Poofery, Osamu Kaneko, Masahito Asada, Morakot Kaewthamasorn

**Affiliations:** 1 The International Graduate Program of Veterinary Science and Technology (VST), Faculty of Veterinary Science, Chulalongkorn University, Bangkok, 10330 Thailand; 2 Veterinary Pathobiology Graduate Program, Faculty of Veterinary Science, Chulalongkorn University, Bangkok, 10330 Thailand; 3 Veterinary Parasitology Research Unit, Faculty of Veterinary Science, Chulalongkorn University, Bangkok, 10330 Thailand; 4 Department of Protozoology, Institute of Tropical Medicine (NEKKEN), Nagasaki University, Nagasaki, 852-8523 Japan; 5 National Research Center for Protozoan Diseases, Department of Global Cooperation, Research Unit for Global Infection Control, Obihiro University of Agriculture and Veterinary, Obihiro, 080-8555 Japan; 6 Department of Parasitology, Faculty of Veterinary Medicine, Universitas Gadjah Mada, Yogyakarta, 55281 Indonesia

Correction to: *Scientific Reports* https://doi.org/10.1038/s41598-022-09686-9, published online 06 April 2022

The original version of this Article contained errors in the order of the Figures. Figures 2 and 3 were published as Figures 3 and 2. As a result, the Figure legends were incorrect.

The original Figures 2 and 3 and accompanying legends appear below.

The original Article has been corrected.

**Figure 2:**
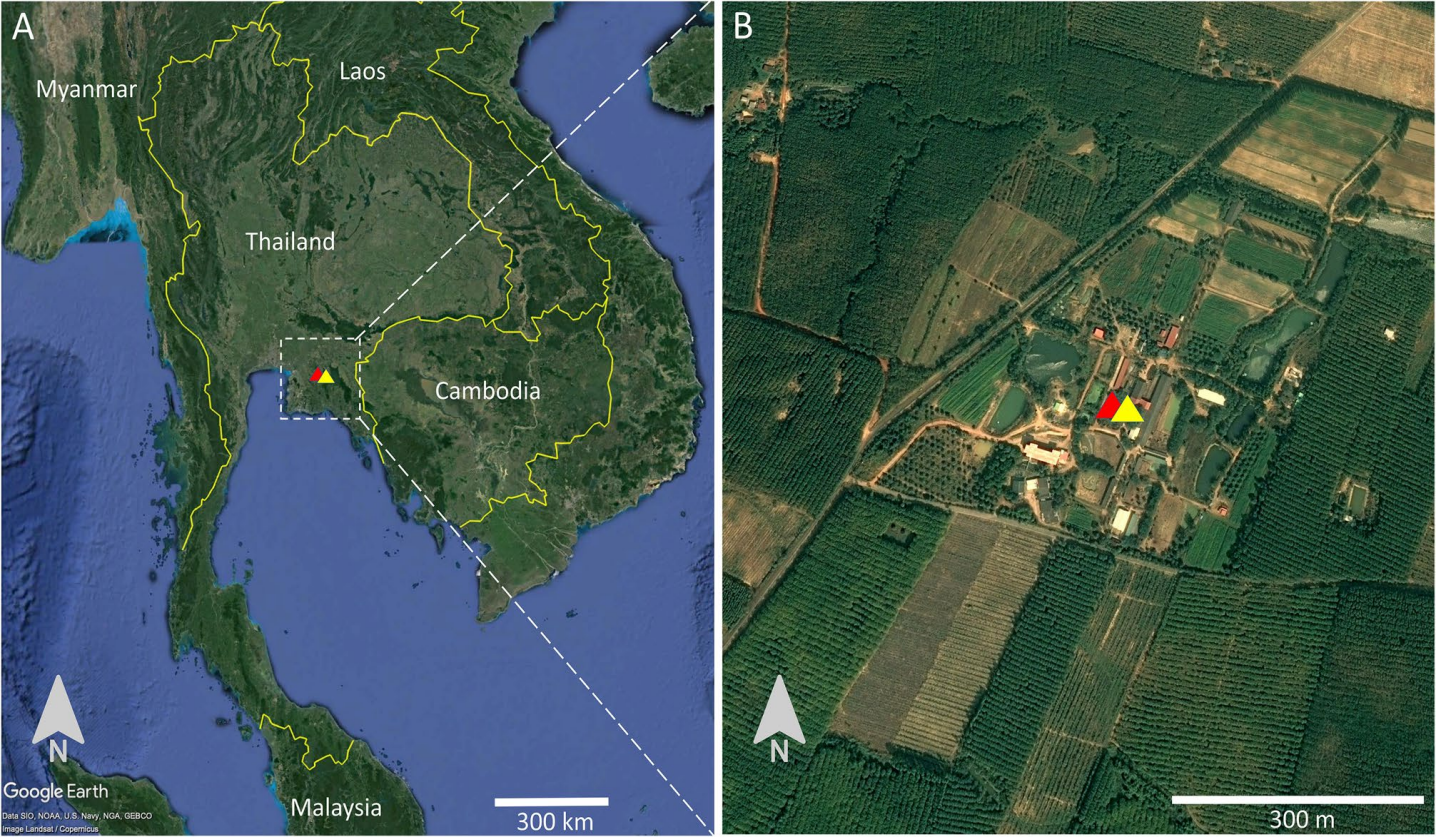
Phylogenetic positions of *Plasmodium* detected from *Anopheles* mosquitoes in this study. The phylogenetic tree was inferred by Bayesian inference method using partial *cytb* sequences (789 bp). *Haemoproteus columbae* was used to root all sequences. At the nodes, Bayesian posterior probabilities (PP ≥ 0.65) are indicated. *Plasmodium* sequences obtained in this study are highlighted in red. The length for the substitutions/site (0.02) is indicated.

**Figure 3:**
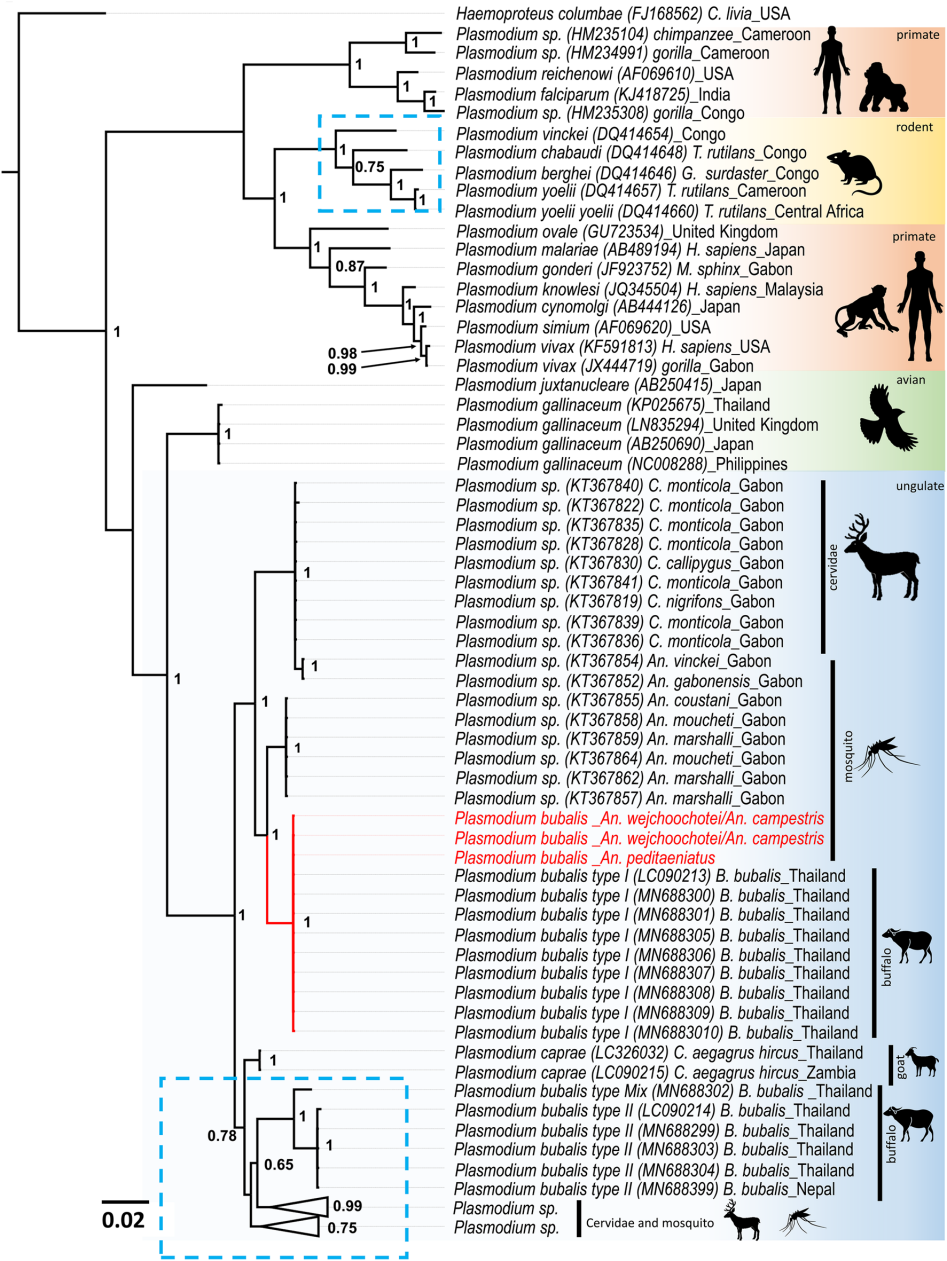
(**A**) Map depicting a buffalo farm in Chachoengsao for sample collection in Thailand. (**B**) The landscape of mosquito sampling sites in a buffalo farm in Chachoengsao. The images were obtained and modified from Google Earth Pro version 7.3.4.8248. The red triangle indicates blood sample collection sites, while the yellow triangle indicates mosquito sampling sites.

