# Supplementary material for: Myzorhynchus series of Anopheles mosquitoes as potential vectors of Plasmodium bubalis in Thailand
Source: Sci Rep. 2022 Apr 6;12:5747. doi: 10.1038/s41598-022-09686-9 (PMC8987089; doi:10.1038/s41598-022-09686-9)
Supplement: Supplementary file 1 — Supplementary Legends. [file 41598_2022_9686_MOESM1_ESM.docx]

**Additional information**

**Supplementary Table 1.** PCR cycling conditions.

**Supplementary Table 2.** Overview of BLASTN results of anopheline mosquitoes in this study.

**Supplementary Fig. 1.** Multiple sequence alignment of cytochrome c oxidase subunit I (*cox1*) gene of *An. wejchoochotei*, and 9 sequences of *Anopheles* spp. obtained from the present study. Mismatch nucleotides are masked.

**Supplementary Fig. 2.** Phylogenetic positions of *Plasmodium* detected from mosquitoes in this study. The Phylogenetic tree was inferred by the Bayesian inference method using partial cox1 gene sequences (254 bp). Bayesian posterior probability values are shown in each node (PP ≥ 0.75). *Plasmodium* sequences obtained in this study are highlighted in red. The length for the substitutions/site (1.0) is indicated.

**Supplementary Fig. 3.** Phylogenetic positions of *Plasmodium* detected from mosquitoes in this study. The phylogenetic tree was inferred by the Bayesian inference method using partial 18S rRNA sequences (351 bp). Bayesian posterior probability values are presented in each node (PP ≥ 0.50). *Plasmodium* sequences obtained in this study are in red color. The length for the substitutions/site (0.02) is indicated.
