# Supplementary material for: Myzorhynchus series of Anopheles mosquitoes as potential vectors of Plasmodium bubalis in Thailand
Source: Sci Rep. 2022 Apr 6;12:5747. doi: 10.1038/s41598-022-09686-9 (PMC8987089; doi:10.1038/s41598-022-09686-9)
Supplement: Supplementary file 2 — Supplementary Figure 1. [file 41598_2022_9686_MOESM2_ESM.docx]

**THMosqBuff_P8_2_*cox1*** ------------------------------------------------------------ 0

THMosqBuff_P3_1_*cox1* GCTTGAGCCGGAATAGTAGGAACTTCTTTAAGTATTCTTATTCGAGCTGAATTAGGTCAT 60

THMosqBuff_P12_1_*cox1* GCTTGAGCCGGAATAGTAGGAACTTCTTTAAGTATTCTTATTCGAGCTGAATTAGGTCAT 60

THMosqBuff_P1_1_*cox1* GCTTGAGCCGGAATAGTAGGAACTTCTTTAAGTATTCTTATTCGAGCTGAATTAGGTCAT 60

**THMosqBuff_P6_3_*cox1*** GCTTGAGCCGGAATAGTAGGAACTTCTTTAAGTATTCTTATTCGAGCTGAATTAGGTCAT 60

THMosqBuff_P25_1_*cox1* GCTTGAGCCGGAATAGTAGGAACTTCTTTAAGTATTCTTATTCGAGCTGAATTAGGTCAT 60

THMosqBuff_P26_1_*cox1* GCTTGAGCCGGAATAGTAGGAACTTCTTTAAGTATTCTTATTCGAGCTGAATTAGGTCAT 60

THMosqBuff_P29_1_*cox1* GCTTGAGCCGGAATAGTAGGAACTTCTTTAAGTATTCTTATTCGAGCTGAATTAGGTCAT 60

THMosqBuff_P30_2_*cox1* GCTTGAGCCGGAATAGTAGGAACTTCTTTAAGTATTCTTATTCGAGCTGAATTAGGTCAT 60

AB971337_*An.wejchoochotei*_*cox1* GCTTGAGCCGGAATAGTAGGAACTTCTTTAAGTATTCTTATTCGAGCTGAATTAGGTCAT 60

AB971335_*An.wejchoochotei*_*cox1* GCTTGAGCCGGAATAGTAGGAACTTCTTTAAGTATTCTTATTCGAGCTGAATTAGGTCAT 60

AB971340_*An.wejchoochotei*_*cox1* GCTTGAGCCGGAATAGTAGGAACTTCTTTAAGTATTCTTATTCGAGCTGAATTAGGTCAT 60

AB971336_*An.wejchoochotei*_*cox1* GCTTGAGCCGGAATAGTAGGAACTTCTTTAAGTATTCTTATTCGAGCTGAATTAGGTCAT 60

AB971338_*An.wejchoochotei*_*cox1* GCTTGAGCCGGAATAGTAGGAACTTCTTTAAGTATTCTTATTCGAGCTGAATTAGGTCAT 60

AB971339_*An.wejchoochotei*_*cox1* GCTTGAGCCGGAATAGTAGGAACTTCTTTAAGTATTCTTATTCGAGCTGAATTAGGTCAT 60

************************************************************

**THMosqBuff_P8_2_*cox1*** ------------------------------------------------------------ 0

THMosqBuff_P3_1_*cox1* CCAGGAGCTTTTATTGGAGATGATCAAATTTATAATGTAATTGTTACAGCTCATGCTTTT 120

THMosqBuff_P12_1_*cox1* CCAGGAGCTTTTATTGGAGATGATCAAATTTATAATGTAATTGTTACAGCTCATGCTTTT 120

THMosqBuff_P1_1_*cox1* CCAGGAGCTTTTATTGGAGATGATCAAATTTATAATGTAATTGTTACAGCTCATGCTTTT 120

**THMosqBuff_P6_3_*cox1*** CCAGGAGCTTTTATTGGAGATGATCAAATTTATAATGTAATTGTTACAGCTCATGCTTTT 120

THMosqBuff_P25_1_*cox1* CCAGGAGCTTTTATTGGAGATGATCAAATTTATAATGTAATTGTTACAGCTCATGCTTTT 120

THMosqBuff_P26_1_*cox1* CCAGGAGCTTTTATTGGAGATGATCAAATTTATAATGTAATTGTTACAGCTCATGCTTTT 120

THMosqBuff_P29_1_*cox1* CCAGGAGCTTTTATTGGAGATGATCAAATTTATAATGTAATTGTTACAGCTCATGCTTTT 120

THMosqBuff_P30_2_*cox1* CCAGGAGCTTTTATTGGAGATGATCAAATTTATAATGTAATTGTTACAGCTCATGCTTTT 120

AB971337_*An.wejchoochotei*_*cox1* CCAGGAGCTTTTATTGGAGATGATCAAATTTATAATGTAATTGTTACAGCTCATGCTTTT 120

AB971335_*An.wejchoochotei*_*cox1* CCAGGAGCTTTTATTGGAGATGATCAAATTTATAATGTAATTGTTACAGCTCATGCTTTT 120

AB971340_*An.wejchoochotei*_*cox1* CCAGGAGCTTTTATTGGAGATGATCAAATTTATAATGTAATTGTTACAGCTCATGCTTTT 120

AB971336_*An.wejchoochotei*_*cox1* CCAGGAGCTTTTATTGGAGATGATCAAATTTATAATGTAATTGTTACAGCTCATGCTTTT 120

AB971338_*An.wejchoochotei*_*cox1* CCAGGAGCTTTTATTGGAGATGATCAAATTTATAATGTAATTGTTACAGCTCATGCTTTT 120

AB971339_*An.wejchoochotei*_*cox1* CCAGGAGCTTTTATTGGAGATGATCAAATTTATAATGTAATTGTTACAGCTCATGCTTTT 120

************************************************************

**THMosqBuff_P8_2_*cox1*** ------------------------------------------------------------ 0

THMosqBuff_P3_1_*cox1* ATTATAATTTTCTTTATAGTTATACCTATTATAATTGGAGGATTTGGAAACTGATTAGTG 180

THMosqBuff_P12_1_*cox1* ATTATAATTTTCTTTATAGTTATACCTATTATAATTGGAGGATTTGGAAACTGATTAGTG 180

THMosqBuff_P1_1_*cox1* ATTATAATTTTCTTTATAGTTATACCTATTATAATTGGAGGATTTGGAAACTGATTAGTG 180

**THMosqBuff_P6_3_*cox1*** ATTATAATTTTCTTTATAGTTATACCTATTATAATTGGAGGATTTGGAAACTGATTAGTG 180

THMosqBuff_P25_1_*cox1* ATTATAATTTTCTTTATAGTTATACCTATTATAATTGGAGGATTTGGAAACTGATTAGTG 180

THMosqBuff_P26_1_*cox1* ATTATAATTTTCTTTATAGTTATACCTATTATAATTGGAGGATTTGGAAACTGATTAGTG 180

THMosqBuff_P29_1_*cox1* ATTATAATTTTCTTTATAGTTATACCTATTATAATTGGAGGATTTGGAAACTGATTAGTG 180

THMosqBuff_P30_2_*cox1* ATTATAATTTTCTTTATAGTTATACCTATTATAATTGGAGGATTTGGAAACTGATTAGTG 180

AB971337_*An.wejchoochotei*_*cox1* ATTATAATTTTCTTTATAGTTATACCTATTATAATTGGAGGATTTGGAAACTGATTAGTG 180

AB971335_*An.wejchoochotei*_*cox1* ATTATAATTTTCTTTATAGTCATACCTATTATAATTGGAGGATTTGGAAACTGATTAGTG 180

AB971340_*An.wejchoochotei*_*cox1* ATTATAATTTTCTTTATAGTTATACCTATTATAATTGGAGGATTTGGAAACTGATTAGTG 180

AB971336_*An.wejchoochotei*_*cox1* ATTATAATTTTCTTTATAGTTATACCTATTATAATTGGAGGATTTGGAAACTGATTAGTG 180

AB971338_*An.wejchoochotei*_*cox1* ATTATAATTTTCTTTATAGTTATACCTATTATAATTGGAGGATTTGGAAACTGATTAGTG 180

AB971339_*An.wejchoochotei*_*cox1* ATTATAATTTTCTTTATAGTTATACCTATTATAATTGGAGGATTTGGAAACTGATTAGTG 180

******************** ***************************************

**THMosqBuff_P8_2_*cox1*** ------------------------------------------------------------ 0

THMosqBuff_P3_1_*cox1* CCTTTAATATTAGGAGCTCCTGATATAGCATTTCCTCGAATAAATAATATAAGATTTTGA 240

THMosqBuff_P12_1_*cox1* CCTTTAATATTAGGAGCTCCTGATATAGCATTTCCTCGAATAAATAATATAAGATTTTGA 240

THMosqBuff_P1_1_*cox1* CCTTTAATATTAGGAGCTCCTGATATAGCATTTCCTCGAATAAATAATATAAGATTTTGA 240

**THMosqBuff_P6_3_*cox1*** CCTTTAATATTAGGAGCTCCTGATATAGCATTTCCTCGAATAAATAATATAAGATTTTGA 240

THMosqBuff_P25_1_*cox1* CCTTTAATATTAGGAGCTCCTGATATAGCATTTCCTCGAATAAATAATATAAGATTTTGA 240

THMosqBuff_P26_1_*cox1* CCTTTAATATTAGGAGCTCCTGATATAGCATTTCCTCGAATAAATAATATAAGATTTTGA 240

THMosqBuff_P29_1_*cox1* CCTTTAATATTAGGAGCTCCTGATATAGCATTTCCTCGAATAAATAATATAAGATTTTGA 240

THMosqBuff_P30_2_*cox1* CCTTTAATATTAGGAGCTCCTGATATAGCATTTCCTCGAATAAATAATATAAGATTTTGA 240

AB971337_*An.wejchoochotei*_*cox1* CCTTTAATATTAGGAGCTCCTGATATAGCATTTCCTCGAATAAATAATATAAGATTTTGA 240

AB971335_*An.wejchoochotei*_*cox1* CCTTTAATATTAGGAGCTCCTGATATAGCATTTCCTCGAATAAATAATATAAGATTTTGA 240

AB971340_*An.wejchoochotei*_*cox1* CCTTTAATATTAGGAGCTCCTGATATAGCATTTCCTCGAATAAATAATATAAGATTTTGA 240

AB971336_*An.wejchoochotei*_*cox1* CCTTTAATATTAGGAGCTCCTGATATAGCATTTCCTCGAATAAATAATATAAGATTTTGA 240

AB971338_*An.wejchoochotei*_*cox1* CCTTTAATATTAGGAGCTCCTGATATAGCATTTCCTCGAATAAATAATATAAGATTTTGA 240

AB971339_*An.wejchoochotei*_*cox1* CCTTTAATATTAGGAGCTCCTGATATAGCATTTCCTCGAATAAATAATATAAGATTTTGA 240

************************************************************

**THMosqBuff_P8_2_*cox1*** ------------------ACTTTATTAATTTCTAGAAGTATAGTAGAAAATGGGGCTGGA 42

THMosqBuff_P3_1_*cox1* ATATTACCTCCTTCTCTTACTTTATTAATTTCTAGAAGTATAGTAGAAAATGGGGCTGGA 300

THMosqBuff_P12_1_*cox1* ATATTACCTCCTTCTCTTACTTTATTAATTTCTAGAAGTATAGTAGAAAATGGGGCTGGA 300

THMosqBuff_P1_1_*cox1* ATATTACCTCCTTCTCTTACTTTATTAATTTCTAGAAGTATAGTAGAAAATGGGGCTGGA 300

**THMosqBuff_P6_3_*cox1*** ATATTACCTCCTTCTCTTACTTTATTAATTTCTAGAAGTATAGTAGAAAATGGGGCTGGA 300

THMosqBuff_P25_1_*cox1* ATATTACCTCCTTCTCTTACTTTATTAATTTCTAGAAGTATAGTAGAAAATGGGGCTGGA 300

THMosqBuff_P26_1_*cox1* ATATTACCTCCTTCTCTTACTTTATTAATTTCTAGAAGTATAGTAGAAAATGGGGCTGGA 300

THMosqBuff_P29_1_*cox1* ATATTACCTCCTTCTCTTACTTTATTAATTTCTAGAAGTATAGTAGAAAATGGGGCTGGA 300

THMosqBuff_P30_2_*cox1* ATATTACCTCCTTCTCTTACTTTATTAATTTCTAGAAGTATAGTAGAAAATGGGGCTGGA 300

AB971337_*An.wejchoochotei*_*cox1* ATATTACCTCCTTCTCTTACTTTATTAATTTCTAGAAGTATAGTAGAAAATGGGGCTGGA 300

AB971335_*An.wejchoochotei*_*cox1* ATATTACCTCCTTCTCTTACTTTATTAATTTCTAGAAGTATAGTAGAAAATGGGGCTGGA 300

AB971340_*An.wejchoochotei*_*cox1* ATATTACCTCCTTCTCTTACTTTATTAATTTCTAGAAGTATAGTAGAAAATGGGGCTGGA 300

AB971336_*An.wejchoochotei*_*cox1* ATATTACCTCCTTCTCTTACTTTATTAATTTCTAGAAGTATAGTAGAAAATGGGGCTGGA 300

AB971338_*An.wejchoochotei*_*cox1* ATATTACCTCCTTCTCTTACTTTATTAATTTCTAGAAGTATAGTAGAAAATGGGGCTGGA 300

AB971339_*An.wejchoochotei*_*cox1* ATATTACCTCCTTCTCTTACTTTATTAATTTCTAGAAGTATAGTAGAAAATGGGGCTGGA 300

************************************************************

**THMosqBuff_P8_2_*cox1*** ACTGGGTGAACTGTTTACCCTCCTTTATCTTCTGGGATTGCTCATGCAGGAGCTTCTGTT 102

THMosqBuff_P3_1_*cox1* ACTGGGTGAACTGTTTACCCTCCTTTATCTTCTGGGATTGCTCATGCAGGAGCTTCTGTT 360

THMosqBuff_P12_1_*cox1* ACTGGGTGAACTGTTTACCCTCCTTTATCTTCTGGGATTGCTCATGCAGGAGCTTCTGTT 360

THMosqBuff_P1_1_*cox1* ACTGGGTGAACTGTTTACCCTCCTTTATCTTCTGGGATTGCTCATGCAGGAGCTTCTGTT 360

**THMosqBuff_P6_3_*cox1*** ACTGGGTGAACTGTTTACCCTCCTTTATCTTCTGGGATTGCTCATGCAGGAGCTTCTGTT 360

THMosqBuff_P25_1_*cox1* ACTGGGTGAACTGTTTACCCTCCTTTATCTTCTGGGATTGCTCATGCAGGAGCTTCTGTT 360

THMosqBuff_P26_1_*cox1* ACTGGGTGAACTGTTTACCCTCCTTTATCTTCTGGGATTGCTCATGCAGGAGCTTCTGTT 360

THMosqBuff_P29_1_*cox1* ACTGGGTGAACTGTTTACCCTCCTTTATCTTCTGGGATTGCTCATGCAGGAGCTTCTGTT 360

THMosqBuff_P30_2_*cox1* ACTGGGTGAACTGTTTACCCTCCTTTATCTTCTGGGATTGCTCATGCAGGAGCTTCTGTT 360

AB971337_*An.wejchoochotei*_*cox1* ACTGGATGAACTGTTTACCCTCCTTTATCTTCTGGGATTGCTCATGCAGGAGCTTCTGTT 360

AB971335_*An.wejchoochotei*_*cox1* ACTGGATGAACTGTTTACCCTCCTTTATCTTCTGGGATTGCTCATGCAGGAGCTTCTGTT 360

AB971340_*An.wejchoochotei*_*cox1* ACTGGATGAACTGTTTACCCTCCTTTATCTTCTGGGATTGCTCATGCAGGAGCTTCTGTT 360

AB971336_*An.wejchoochotei*_*cox1* ACTGGATGAACTGTTTACCCTCCTTTATCTTCTGGGATTGCTCATGCAGGAGCTTCTGTT 360

AB971338_*An.wejchoochotei*_*cox1* ACTGGATGAACTGTTTACCCTCCTTTATCTTCTGGGATTGCTCATGCAGGAGCTTCTGTT 360

AB971339_*An.wejchoochotei*_*cox1* ACTGGATGAACTGTTTACCCTCCTTTATCTTCTGGGATTGCTCATGCAGGAGCTTCTGTT 360

***** ******************************************************

**THMosqBuff_P8_2_*cox1*** GATTTAGCAATTTTTTCATTACATTTAGCAGGAATTTCTTCAATTTTAGGAGCAGTAAAT 162

THMosqBuff_P3_1_*cox1* GATTTAGCAATTTTTTCATTACATTTAGCAGGAATTTCTTCAATTTTAGGAGCAGTAAAT 420

THMosqBuff_P12_1_*cox1* GATTTAGCAATTTTTTCATTACATTTAGCAGGAATTTCTTCAATTTTAGGAGCAGTAAAT 420

THMosqBuff_P1_1_*cox1* GATTTAGCAATTTTTTCATTACATTTAGCAGGAATTTCTTCAATTTTAGGAGCAGTAAAT 420

**THMosqBuff_P6_3_*cox1*** GATTTAGCAATTTTTTCATTACATTTAGCAGGAATTTCTTCAATTTTAGGAGCAGTAAAT 420

THMosqBuff_P25_1_*cox1* GATTTAGCAATTTTTTCATTACATTTAGCAGGAATTTCTTCAATTTTAGGAGCAGTAAAT 420

THMosqBuff_P26_1_*cox1* GATTTAGCAATTTTTTCATTACATTTAGCAGGAATTTCTTCAATTTTAGGAGCAGTAAAT 420

THMosqBuff_P29_1_*cox1* GATTTAGCAATTTTTTCATTACATTTAGCAGGAATTTCTTCAATTTTAGGAGCAGTAAAT 420

THMosqBuff_P30_2_*cox1* GATTTAGCAATTTTTTCATTACATTTAGCAGGAATTTCTTCAATTTTAGGAGCAGTAAAT 420

AB971337_*An.wejchoochotei*_*cox1* GATTTAGCAATTTTTTCATTACATTTAGCAGGAATTTCTTCAATTTTAGGAGCAGTAAAT 420

AB971335_*An.wejchoochotei*_*cox1* GATTTAGCAATTTTTTCATTACATTTAGCAGGAATTTCTTCAATTTTAGGAGCAGTAAAT 420

AB971340_*An.wejchoochotei*_*cox1* GATTTAGCAATTTTTTCATTACATTTAGCAGGAATTTCTTCAATTTTAGGAGCAGTAAAT 420

AB971336_*An.wejchoochotei*_*cox1* GATTTAGCAATTTTTTCATTACATTTAGCAGGAATTTCTTCAATTTTAGGAGCAGTAAAT 420

AB971338_*An.wejchoochotei*_*cox1* GATTTAGCAATTTTTTCATTACATTTAGCAGGAATTTCTTCAATTTTAGGAGCAGTAAAT 420

AB971339_*An.wejchoochotei*_*cox1* GATTTAGCAATTTTTTCATTACATTTAGCAGGAATTTCTTCAATTTTAGGAGCAGTAAAT 420

************************************************************

**THMosqBuff_P8_2_*cox1*** TTTATTACTACTGTTATTAATATACGTTCACCAGGAATTACTCTTGATCGAATACCTTTA 222

THMosqBuff_P3_1_*cox1* TTTATTACTACTGTTATTAATATACGTTCACCAGGAATTACTCTTGATCGAATACCTTTA 480

THMosqBuff_P12_1_*cox1* TTTATTACTACTGTTATTAATATACGTTCACCAGGAATTACTCTTGATCGAATACCTTTA 480

THMosqBuff_P1_1_*cox1* TTTATTACTACTGTTATTAATATACGTTCACCAGGAATTACTCTTGATCGAATACCTTTA 480

**THMosqBuff_P6_3_*cox1*** TTTATTACTACTGTTATTAATATACGTTCACCAGGAATTACTCTTGATCGAATACCTTTA 480

THMosqBuff_P25_1_*cox1* TTTATTACTACTGTTATTAATATACGTTCACCAGGAATTACTCTTGATCGAATACCTTTA 480

THMosqBuff_P26_1_*cox1* TTTATTACTACTGTTATTAATATACGTTCACCAGGAATTACTCTTGATCGAATACCTTTA 480

THMosqBuff_P29_1_*cox1* TTTATTACTACTGTTATTAATATACGTTCACCAGGAATTACTCTTGATCGAATACCTTTA 480

THMosqBuff_P30_2_*cox1* TTTATTACTACTGTTATTAATATACGTTCACCAGGAATTACTCTTGATCGAATACCTTTA 480

AB971337_*An.wejchoochotei*_*cox1* TTTATTACTACTGTTATTAATATACGTTCACCAGGAATTACTCTTGATCGAATACCTTTA 480

AB971335_*An.wejchoochotei*_*cox1* TTTATTACTACTGTTATTAATATACGTTCACCAGGAATTACTCTTGATCGAATACCTTTA 480

AB971340_*An.wejchoochotei*_*cox1* TTTATTACTACTGTTATTAATATACGTTCACCAGGAATTACTCTTGATCGAATACCTTTA 480

AB971336_*An.wejchoochotei*_*cox1* TTTATTACTACTGTTATTAATATACGTTCACCAGGAATTACTCTTGATCGAATACCTTTA 480

AB971338_*An.wejchoochotei*_*cox1* TTTATTACTACTGTTATTAATATACGTTCACCAGGAATTACTCTTGATCGAATACCTTTA 480

AB971339_*An.wejchoochotei*_*cox1* TTTATTACTACTGTTATTAATATACGTTCACCAGGAATTACTCTTGATCGAATACCTTTA 480

************************************************************

**THMosqBuff_P8_2_*cox1*** TTTGTTTGATCTGTAGTTATTACAGCAGTTCTTTTATTATTATCTTTACCAGTGTTAGCA 282

THMosqBuff_P3_1_*cox1* TTTGTTTGATCTGTAGTTATTACAGCAGTTCTTTTATTATTATCTTTACCAGTGTTAGCA 540

THMosqBuff_P12_1_*cox1* TTTGTTTGATCTGTAGTTATTACAGCAGTTCTTTTATTATTATCTTTACCAGTGTTAGCA 540

THMosqBuff_P1_1_*cox1* TTTGTTTGATCTGTAGTTATTACAGCAGTTCTTTTATTATTATCTTTACCAGTGTTAGCA 540

**THMosqBuff_P6_3_*cox1*** TTTGTTTGATCTGTAGTTATTACAGCAGTTCTTTTATTATTATCTTTACCAGTGTTAGCA 540

THMosqBuff_P25_1_*cox1* TTTGTTTGATCTGTAGTTATTACAGCAGTTCTTTTATTATTATCTTTACCAGTGTTAGCA 540

THMosqBuff_P26_1_*cox1* TTTGTTTGATCTGTAGTTATTACAGCAGTTCTTTTATTATTATCTTTACCAGTGTTAGCA 540

THMosqBuff_P29_1_*cox1* TTTGTTTGATCTGTAGTTATTACAGCAGTTCTTTTATTATTATCTTTACCAGTGTTAGCA 540

THMosqBuff_P30_2_*cox1* TTTGTTTGATCTGTAGTTATTACAGCAGTTCTTTTATTATTATCTTTACCAGTGTTAGCA 540

AB971337_*An.wejchoochotei*_*cox1* TTTGTTTGATCTGTAGTTATTACAGCAGTTCTTTTATTATTATCTTTACCAGTGTTAGCA 540

AB971335_*An.wejchoochotei*_*cox1* TTTGTTTGATCTGTAGTTATTACAGCAGTTCTTTTATTATTATCTTTACCAGTGTTAGCA 540

AB971340_*An.wejchoochotei*_*cox1* TTTGTTTGATCTGTAGTTATTACAGCAGTTCTTTTATTATTATCTTTACCAGTGTTAGCA 540

AB971336_*An.wejchoochotei*_*cox1* TTTGTTTGATCTGTAGTTATTACAGCAGTTCTTTTATTATTATCTTTACCAGTGTTAGCA 540

AB971338_*An.wejchoochotei*_*cox1* TTTGTTTGATCTGTAGTTATTACAGCAGTTCTTTTATTATTATCTTTACCAGTGTTAGCA 540

AB971339_*An.wejchoochotei*_*cox1* TTTGTTTGATCTGTAGTTATTACAGCAGTTCTTTTATTATTATCTTTACCAGTGTTAGCA 540

************************************************************

**THMosqBuff_P8_2_*cox1*** GGAGCAATTACTATATTATTAACTGATCGAAATTTAAATACATCTTTCTTT--------- 333

THMosqBuff_P3_1_*cox1* GGAGCAATTACTATATTATTAACTGATCGAAATTTAAATACATCTTTCTTTGACCCTGCA 600

THMosqBuff_P12_1_*cox1* GGAGCAATTACTATATTATTAACTGATCGAAATTTAAATACATCTTTCTTTGACCCTGCA 600

THMosqBuff_P1_1_*cox1* GGAGCAATTACTATATTATTAACTGATCGAAATTTAAATACATCTTTCTTTGACCCTGCA 600

**THMosqBuff_P6_3_*cox1*** GGAGCAATTACTATATTATTAACTGATCGAAATTTAAATACATCTTTCTTTGACCCTGCA 600

THMosqBuff_P25_1_*cox1* GGAGCAATTACTATATTATTAACTGATCGAAATTTAAATACATCTTTCTTTGACCCTGCA 600

THMosqBuff_P26_1_*cox1* GGAGCAATTACTATATTATTAACTGATCGAAATTTAAATACATCTTTCTTTGACCCTGCA 600

THMosqBuff_P29_1_*cox1* GGAGCAATTACTATATTATTAACTGATCGAAATTTAAATACATCTTTCTTTGACCCTGCA 600

THMosqBuff_P30_2_*cox1* GGAGCAATTACTATATTATTAACTGATCGAAATTTAAATACATCTTTCTTTGACCCTGCA 600

AB971337_*An.wejchoochotei*_*cox1* GGAGCAATTACTATATTATTAACTGATCGAAATTTAAATACATCTTTCTTTGACCCTGCA 600

AB971335_*An.wejchoochotei*_*cox1* GGAGCAATTACTATATTATTAACTGATCGAAATTTAAATACATCTTTCTTTGACCCTGCA 600

AB971340_*An.wejchoochotei*_*cox1* GGAGCAATTACTATATTATTAACTGATCGAAATTTAAATACATCTTTCTTTGACCCTGCA 600

AB971336_*An.wejchoochotei*_*cox1* GGAGCAATTACTATATTATTAACTGATCGAAATTTAAATACATCTTTCTTTGACCCTGCA 600

AB971338_*An.wejchoochotei*_*cox1* GGAGCAATTACTATATTATTAACTGATCGAAATTTAAATACATCTTTCTTTGACCCTGCA 600

AB971339_*An.wejchoochotei*_*cox1* GGAGCAATTACTATATTATTAACTGATCGAAATTTAAATACATCTTTCTTTGACCCTGCA 600

************************************************************

**THMosqBuff_P8_2_*cox1*** ------------------------------------ 333

THMosqBuff_P3_1_*cox1* GGAGGAGGAGACCCAATTTTATATCAACATTTATTT 636

THMosqBuff_P12_1_*cox1* GGAGGAGGAGACCCAATTTTATATCAACATTTATTT 636

THMosqBuff_P1_1_*cox1* GGAGGAGGAGACCCAATTTTATATCAACATTTATTT 636

**THMosqBuff_P6_3_*cox1*** GGAGGAGGAGACCCAATTTTATATCAACATTTATTT 636

THMosqBuff_P25_1_*cox1* GGAGGAGGAGACCCAATTTTATATCAACATTTATTT 636

THMosqBuff_P26_1_*cox1* GGAGGAGGAGACCCAATTTTATATCAACATTTATTT 636

THMosqBuff_P29_1_*cox1* GGAGGAGGAGATCCAATTTTATATCAACATTTATTT 636

THMosqBuff_P30_2_*cox1* GGAGGAGGAGATCCAATTTTATATCAACATTTATTT 636

AB971337_*An.wejchoochotei*_*cox1* GGAGGAGGAGATCCAATTTTATATCAACATTTATTT 636

AB971335_*An.wejchoochotei*_*cox1* GGAGGAGGAGACCCAATTTTATATCAACATTTATTT 636

AB971340_*An.wejchoochotei*_*cox1* GGAGGAGGAGACCCAATTTTATATCAACATTTATTT 636

AB971336_*An.wejchoochotei*_*cox1* GGAGGAGGAGACCCAATTTTATATCAACATTTATTT 636

AB971338_*An.wejchoochotei*_*cox1* GGAGGAGGAGACCCAATTTTATATCAACATTTATTT 636

AB971339_*An.wejchoochotei*_*cox1* GGAGGAGGAGACCCAATTTTATATCAACATTTATTT 636

*********** ************************

Asterisks indicate identical nucleotides. Regions not covered by THMosqBuff_P8_2_*cox1* were assessed using only remaining sequences and identical nucleotides were indicated with asterisks. *An. wejchoochotei* *cox1* sequences (AB971337, AB971335, AB971340, AB971336, AB971338 and AB971339) are from Taai and Harbach (2015). At nucleotide position 306 after THMosqBuff_P3_1_*cox1*, all sequences in this study showed G, whereas all *An. wejchoochotei cox1* sequences from Taai and Harbach (2015) showed A. Letter in bold indicates *Plasmodium*-positive anopheline mosquitoes (THMosqBuff20_P6_3 and THMosqBuff20_P8_2).
