# Supplementary figures and images for: Myzorhynchus series of Anopheles mosquitoes as potential vectors of Plasmodium bubalis in Thailand
Source: Sci Rep. 2022 Apr 6;12:5747. doi: 10.1038/s41598-022-09686-9 (PMC8987089; doi:10.1038/s41598-022-09686-9)

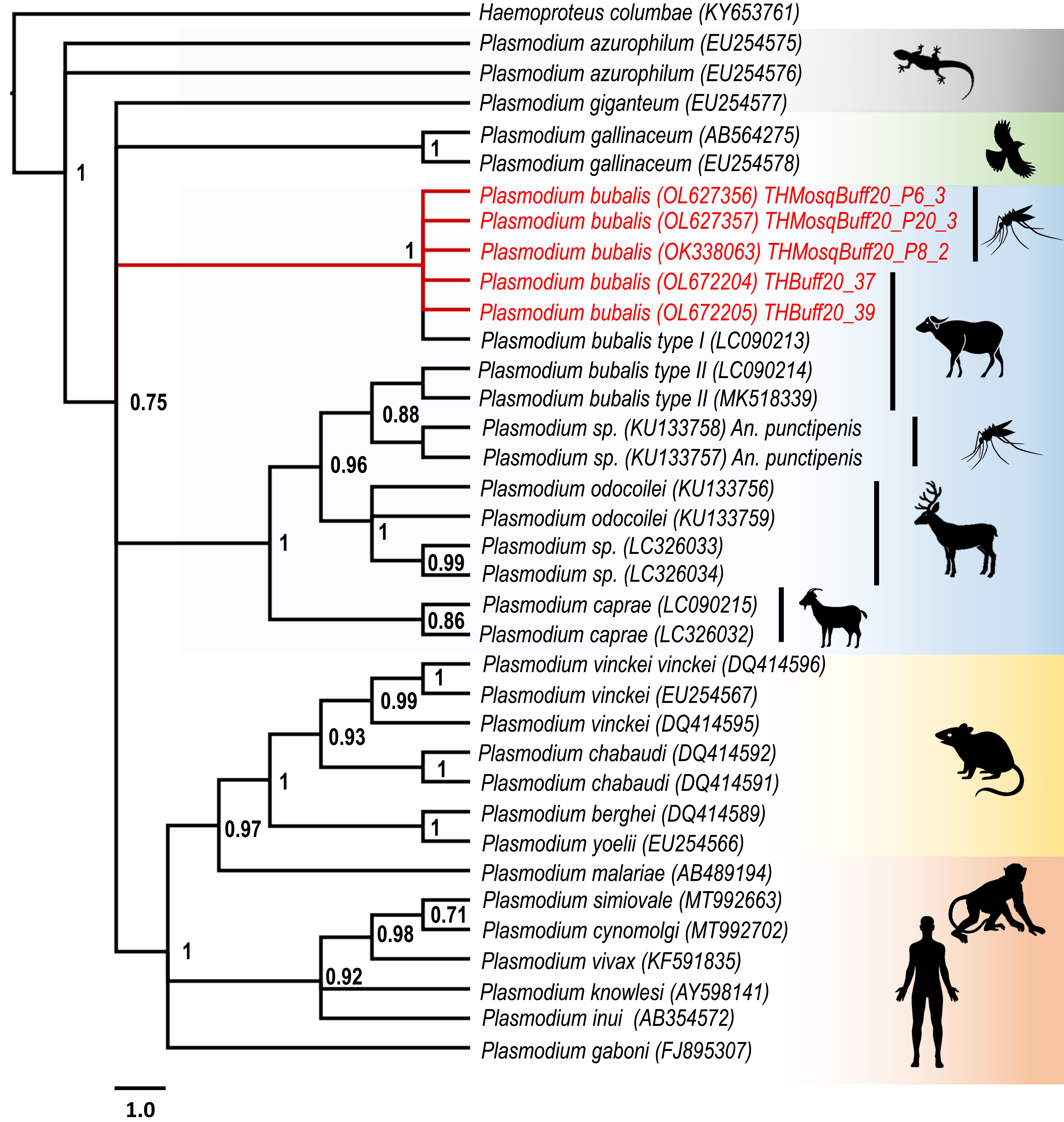

Supplement: Supplementary file 3 — Supplementary Figure 2. [file 41598_2022_9686_MOESM3_ESM.tif]

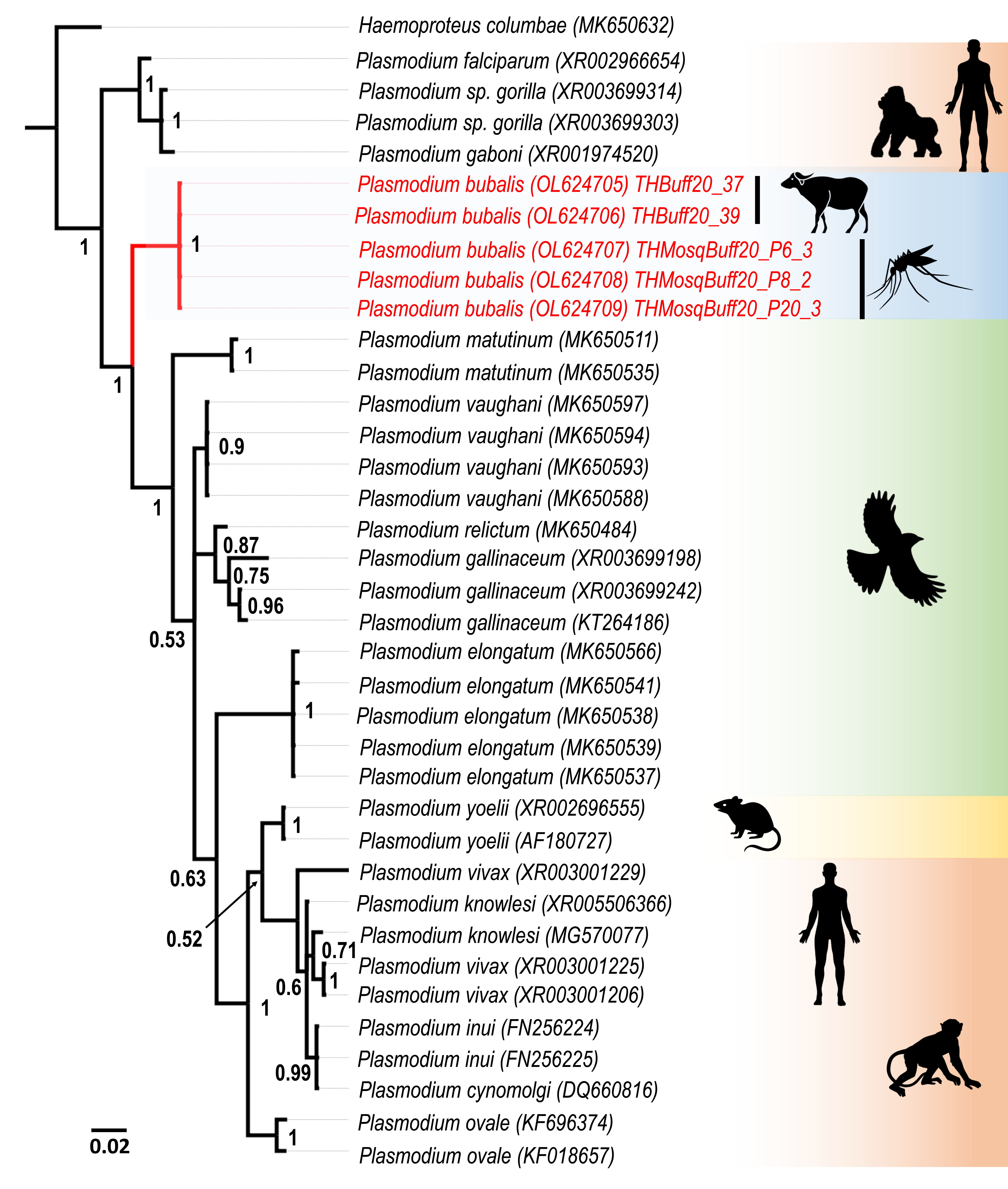

Supplement: Supplementary file 4 — Supplementary Figure 3. [file 41598_2022_9686_MOESM4_ESM.tif]
