# Supplementary material for: Myzorhynchus series of Anopheles mosquitoes as potential vectors of Plasmodium bubalis in Thailand
Source: Sci Rep. 2022 Apr 6;12:5747. doi: 10.1038/s41598-022-09686-9 (PMC8987089; doi:10.1038/s41598-022-09686-9)
Supplement: Supplementary file 5 — Supplementary Table 1. [file 41598_2022_9686_MOESM5_ESM.docx]

**Supplementary Table 1. PCR cycling conditions**

| **Genes***  **(Size)** | **Primer names** | **PCR condition** | **Reference** |
| --- | --- | --- | --- |
| Anopheline mosquito identification | | |  |
| *cox1*  (1,584 bp) | AnplCox1F-AnplCox1R | 94^o^C × 2 min;  **35 cycles: 98^o^C × 20 sec, 56^o^C × 30 sec, 68^o^C × 1 min 40 sec;**  68^o^C × 2 min | This study |
| *cox2*  (792 bp) | AnplCox2F- AnplCox2R | 94^o^C × 2 min;  **35 cycles: 98^o^C ×10 sec, 58^o^C ×30 sec, 68^o^C × 40 sec;**  68^o^C × 5 min | This study |
| ITS2  (555-1,500 bp) | ITS2A - ITS2B | 94^o^C × 2 min;  **40 cycles: 98^o^C × 10 sec, 51^o^C × 50 sec, 68^o^C × 1 min;**  68^o^C × 5 min | Beebe and Saul, 1995^44^ |
| *Plasmodium bubalis* identification | | |  |
| **Genes***  **(Size)** | **Primer names** | **PCR condition** | **Reference** |
| *cytb*  (1,138 bp) | DW2-DW4 (outer) | 94^o^C × 2 min;  **40 cycles: 98^o^C × 10 sec, 62^o^C × 3 min;**  68^o^C × 5 min | Perkins and Schall, 2002^45^ |
| *cytb*  (822 bp) | NCYBINF-NCYBINR  (inner) | 94^o^C × 2 min**;**  **40 cycles: 98^o^C × 10 sec, 62^o^C × 3 min;**  68^o^C × 5 min | Templeton et al., 2016b^3^ |
| *18S rRNA*  (1,200 bp) | rPLU6  rPLU5  (outer) | 94^o^C × 2 min;  **40 cycles: 98^o^C × 10 sec, 55^o^C × 30 sec, 68^o^C × 30 sec;**  68^o^C × 5 min | Snounou et al., 1993^46^ |
| *18S rRNA*  (420 bp) | PlaSSUF1  PlaSSUR1  (inner) | 98^o^C × 5 min;  **35 cycles: 98^o^C × 10 sec, 49^o^C × 1 min, 68^o^C × 1 min;**  68^o^C × 5 min | This study |
| *cox1*  (283 bp) | Cox1-F3-2  PbuCox1-4B3 | 94^o^C × 5 min;  **40 cycles: 98^o^C × 10 sec, 60^o^C × 30 sec, 68^o^C × 20 sec;**  68^o^C × 5 min | This study |

Note: *Abbreviation: *cytb*, cytochrome b; *cox1*, cytochrome c oxidase subunit 1; *cox2*, cytochrome c oxidase subunit 2; ITS2, Internal Transcribed Spacer 2; min, minute(s); sec, second. Letters in bold describes the condition of the pre-denaturation, denaturation, and elongation steps.
