# Supplementary material for: Myzorhynchus series of Anopheles mosquitoes as potential vectors of Plasmodium bubalis in Thailand
Source: Sci Rep. 2022 Apr 6;12:5747. doi: 10.1038/s41598-022-09686-9 (PMC8987089; doi:10.1038/s41598-022-09686-9)
Supplement: Supplementary file 6 — Supplementary Table 2. [file 41598_2022_9686_MOESM6_ESM.docx]

**Supplementary Table 2.** Overview of BLASTN results of anopheline mosquitoes in this study.

| **No** | **Sample ID** | **No. of mosquito** | **Scientific name (identity percentage)** | | |
| --- | --- | --- | --- | --- | --- |
|  |  | **in pool** | ***cox1* (%)** | ***cox2* (%)** | **ITS2 (%)** |
| 1 | THMosqBuff20_P1_1 | 1 | *An. donaldi* (97) | *An. campestris* (100) | *An. campestris* (99.9) |
| 2 | THMosqBuff20_P2_1 | 1 | *An. vagus* (99) | *An. vagus* (100) | *An. vagus* (100) |
| 3 | THMosqBuff20_P3_1 | 1 | *An. donaldi* (97.3) | *An. campestris* (100) | *An. campestris* (99) |
| 4 | THMosqBuff20_P4_1 | 1 | *An. varuna* (99.9) | *An. varuna* (100) | *An. varuna* (99.7) |
| **5** | **THMosqBuff20_P6_3** | **3** | ***An. donaldi* (97)** | ***An. campestris* (100)** | ***An. wejchoochotei* (98.8)*/***  ***An. campestris* (98.8)** |
| **6** | **THMosqBuff20_P8_2** | **2** | ***An. campestris* (99.1)** | ***An. campestris* (100)** | ***An. wejchoochotei* (100)*/***  ***An. campestris* (100)** |
| 7 | THMosqBuff20_P9_3 | 3 | *An. vagus* (98.4) | *An. vagus* (98) | *An. vagus* (100) |
| 8 | THMosqBuff20_P12_1 | 1 | *An. donaldi* (97.3) | *An. campestris* (99) | *An. wejchoochotei* (100)*/*  *An. campestris* (100) |
| 9 | THMosqBuff20_P17_1 | 1 | *An. peditaeniatus* (98.5) | *An. peditaeniatus* (100) | *An. peditaeniatus* (100) |
| **10** | **THMosqBuff20_P20_3** | **3** | ***An. peditaeniatus* (99.2)** | ***An. peditaeniatus* (99)** | ***An. peditaeniatus* (100)** |
| 11 | THMosqBuff20_P22_2 | 2 | *An. vagus* (98) | *An. vagus* (99) | *An. vagus* (100) |
| 12 | THMosqBuff20_P23_2 | 2 | none with identity ≥ 97% | *An. pseudojamesi* (99) | *An. pseudojamesi* (93.3) |
| 13 | THMosqBuff20_P24_1 | 1 | *An. vagus* (98.5) | *An. vagus* (99) | *An. vagus* (100) |
| 14 | THMosqBuff20_P25_1 | 1 | *An. donaldi* (97) | *An. campestris* (99) | *An. wejchoochotei* (98.9)*/*  *An. campestris* (98.9) |
| 15 | THMosqBuff20_P26_1 | 1 | *An. donaldi* (97) | *An. campestris* (100) | *An. wejchoochotei* (99.7)*/*  *An. campestris* (99.7) |
| 16 | THMosqBuff20_P27_2 | 2 | *An. vagus* (98) | *An. vagus* (99) | *An. vagus* (100) |
| 17 | THMosqBuff20_P28_1 | 1 | none with identity ≥ 97% | *An. peditaeniatus* (99) | *An. peditaeniatus* (100) |
| 18 | THMosqBuff20_P29_1 | 1 | *An. donaldi* (97.1) | *An. campestris* (99) | *An. wejchoochotei* (99.9) |
| 19 | THMosqBuff20_P30_2 | 2 | *An. donaldi* (97.1) | *An. campestris* (100) | *An. wejchoochotei* (99.3)/  *An. campestris* (99.4) |

**Note:** Table represents the results of NCBI’s BLASTN anlaysis of mosquitoes’ sequences in this study after removing low-quality sequences trace files. Identity percentage was obtained from GenBank™ database comparison. Some BLASTN anlaysis results in *cox1* gene were unable to reach the threshold similar identity of mosquito (≥97%). Ogola et al. ^1^noted that based on the barcode region, several studies have suggested an evolutionary divergence of 2–3% as a threshold for intraspecific variation. This indicates those species might not as the same species. Furthermore, *An. wejchoochotei* (*An. campestris*-like) and *An. campestris* in the Barbirostris complex group cannot be distinguished by molecular analysis at this moment. There was no *An. wejchoochotei* *cox2* sequences available in the GenBank™. Letter in bold indicates *Plasmodium*-positive anopheline mosquitoes.

**Reference:**

1. Ogola, E.O., Chepkorir, E., Sang, R., Tchouassi, D.P. A previously unreported potential malaria vector in a dry ecology of Kenya. *Parasite. Vectors*. 12, 80. <https://doi.org/10.1186/s13071-019-3332-z> (2019).
